# New Effective Intraoperative Techniques for the Prevention of Coronal Imbalance after Circumferential Minimally Invasive Correction Surgery for Adult Spinal Deformity

**DOI:** 10.3390/jcm12175670

**Published:** 2023-08-31

**Authors:** Masayuki Ishihara, Shinichirou Taniguchi, Naoto Ono, Takashi Adachi, Yoichi Tani, Masaaki Paku, Koki Kawashima, Muneharu Ando, Takanori Saito

**Affiliations:** Department of Orthopedic Surgery, Kansai Medical University, 2-3-1 Shinmachi, Hirakata City 573-1191, Japan; tanigucs@takii.kmu.ac.jp (S.T.); naoto_ono0416@outlook.jp (N.O.); adachita@hirakata.kmu.ac.jp (T.A.); taniyoic@gmail.com (Y.T.); kmu.orthopaedics.pak@gmail.com (M.P.); chelseajoesheva@gmail.com (K.K.); mando@gaia.eonet.ne.jp (M.A.); saitot@takii.kmu.ac.jp (T.S.)

**Keywords:** coronal imbalance, adult spinal deformity, circumferential minimally invasive correction surgery, rod rotation, kickstand rod technique

## Abstract

This study aimed to devise measures and investigate their effect on coronal imbalance (CI) after circumferential minimally invasive correction surgery (CMIS) with lateral lumbar interbody fusion and percutaneous pedicle screw for adult spinal deformity (ASD). A total of 115 patients with ASD who underwent CMIS from the lower thoracic spine to the ilium were included. Patients were stratified based on the distance between the spinous process of the upper instrumented vertebra and central sacrum vertical line (UIV-CSVL) after the first intraoperative rod application into groups P (UIV-CSVL > 10 mm, *n* = 50) and G (UIV-CSVL < 10 mm, *n* = 65). Measures to correct postoperative CI introduced during surgery, preoperative and postoperative UIV-CSVL, and changes in UIV-CSVL after various measures (ΔUIV-CSVL) were investigated in group P. Rod rotation (RR), S2 alar-iliac screw distraction (SD), and kickstand-rod (KR) technique were performed in group P. Group P was further divided into group RR (*n* = 38), group SD (RR and SD) (*n* = 7), and group KR (RR and KR) (*n* = 5); the ΔUIV-CSVLs were 13.9 mm, 20.1 mm, and 24.4 mm in these three groups, respectively. Postoperative C7-CSVL < 10 mm was achieved in all three correction groups. In conclusion, our measures enabled sufficient correction of the UIV-CSVL and are useful for preventing CI after CMIS for ASD.

## 1. Introduction

The population of elderly individuals has risen worldwide, which is accompanied by an increase in the incidence of age-related degenerative diseases. Adult spinal deformity (ASD) causes lower back pain, postural abnormalities, gait disturbances, and visceral disorders such as respiratory disorders and reflux esophagitis [1,2,3]. Therefore, the performance of the activities of daily living and quality of life can be improved by correcting the sagittal and coronal plane using corrective surgery [4,5,6,7]. Conventional corrective surgery is extremely invasive, and perioperative complications are considered a serious problem since the target population comprises older individuals [8,9,10,11]. In recent years, the introduction of lateral lumbar interbody fusion (LLIF) has made ASD surgery less invasive, and studies investigating the role of circumferential minimally invasive correction surgery (CMIS) combined with percutaneous pedicle screw (PPS) have increased gradually. CMIS is reportedly less invasive than conventional surgery for ASD [12,13,14,15,16,17,18]. Although the surgical invasiveness is relatively lower with CMIS, coronal imbalance (CI) after ASD is a serious complication of this procedure [19,20]. Sagittal imbalance can be compensated for by altering the alignment of the pelvis, cervical spine, thoracic spine, and lower limb by the patients themselves; however, compensating for the CI is difficult. Flexion of the contralateral hip and knee can compensate for CI but is uncomfortable for the patient [21]. Moreover, CI causes tilting of the pelvis, which may result in gait disturbance [21]. It is difficult to correct CI during the CMIS procedure because the instrumented screws and rods are not visible, and the coronal bender cannot be used. To the best of our knowledge, none of the previous studies have evaluated measures against CI in CMIS for ASD. We devised innovative methods to correct intraoperative CI and prevent the occurrence of postoperative CI. In this study, we introduced various measures against CI and reported their effects after CMIS for ASD.

## 2. Materials and Methods

**Figure 1:**
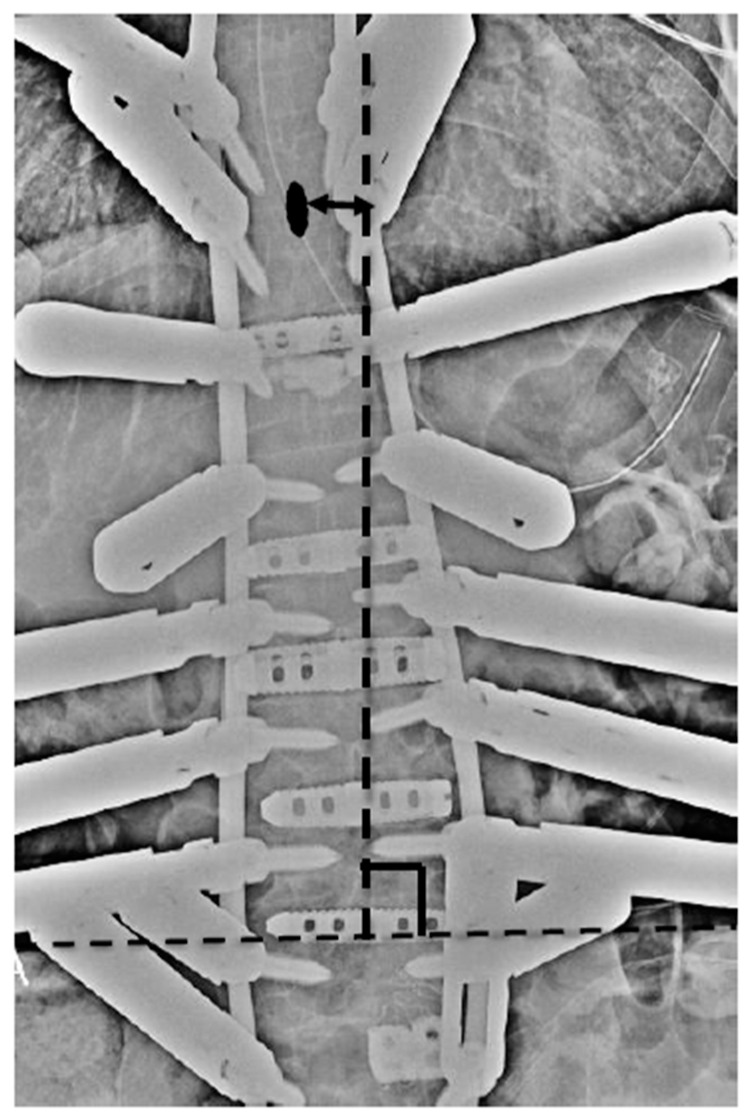
Method of measurement of UIV-CSVL. UIV: upper instrumented vertebra, CSVL: central sacrum vertical line. Black oval: spinous process of UIV.

**Figure 2:**
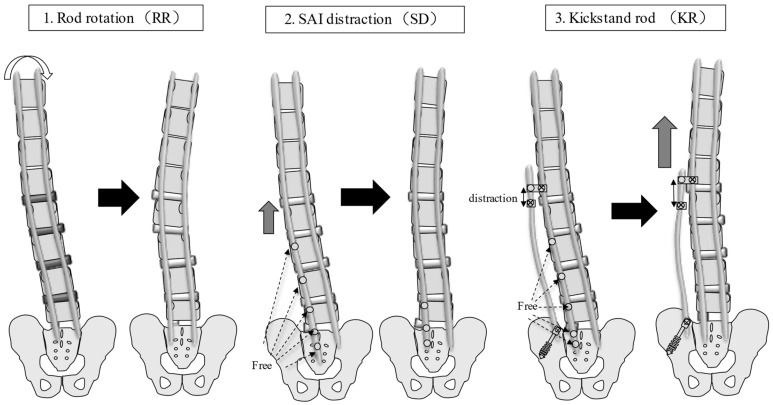
Corrective measures for CI. RR entails the conversion of lumbar lordosis into scoliosis by rotating the two main rods; the main curve remains, and the coronal balance is corrected. SD entails tilting the coronal balance to the opposite side by lifting the main rod on the side, where the coronal balance is tilted cranially using the SAI screw head as a fulcrum. KR entails shifting the coronal balance to the opposite side by shifting the main rod to the cranial side using the additional rod connected to the additional iliac screw as a fulcrum. CI: coronal imbalance; RR: rod rotation technique; SD: sacral alar-iliac screw distraction technique; KR: kickstand rod technique; SAI: sacral alar-iliac screw.

**Figure 3:**
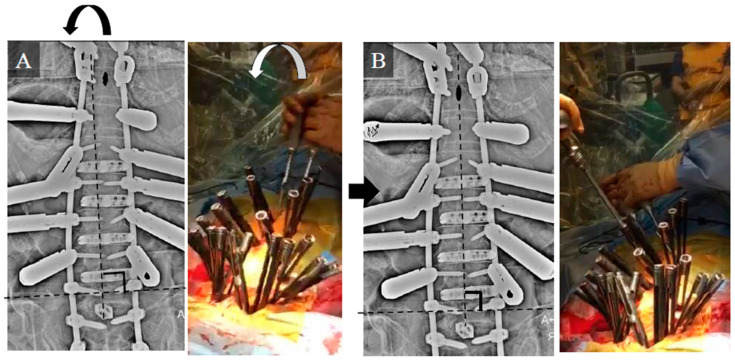
Intraoperative radiograph and photograph of the RR technique. (**A**) Intraoperative radiograph and photograph before RR. (**B**) Intraoperative radiograph and photograph after RR. Black oval: spinous process of the UIV. RR: rod rotation technique; UIV: upper instrumented vertebra.

**Figure 4:**
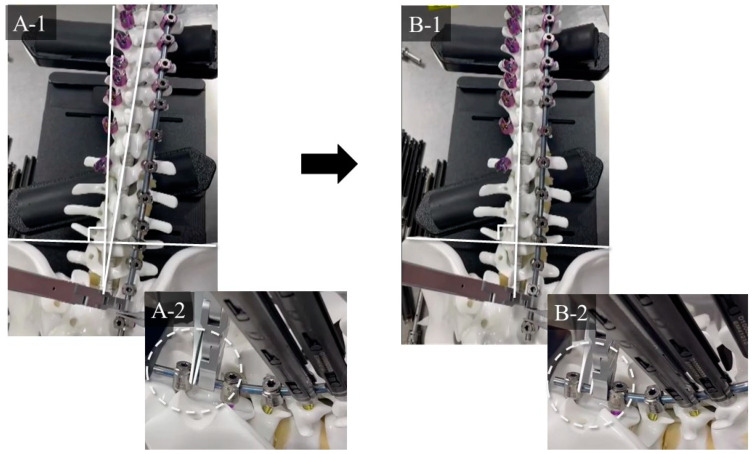
Detailed mechanism of SD. (**A-1**,**A-2**) Before SD. (**B-1**,**B-2**) After SD. By sliding the main rod on the side where the C7-CSVL shifts toward the cranial side, the coronal balance shifts to the opposite side. The rest of the caudal end of the rod is shortened. SD: sacral alar-iliac screw distraction technique; CSVL: central sacrum vertical line.

This study was approved by the Institutional Review Board of Kansai Medical University Hospital (approval number: 2020189; date of approval: 18 January 2021) and was conducted in accordance with the principles of the Declaration of Helsinki. Patients with ASD who underwent CMIS using LLIF and PPS between October 2018 and March 2020 and completed a 24-month postoperative follow-up were evaluated. The inclusion criteria were as follows: patients with ASD, age > 50 years, pelvic incidence (PI)-lumbar lordosis (LL) > 20°, pelvic tilt (PT) > 20°, Cobb angle (CA) > 30°, and fixation from the lower thoracic spine to the pelvis. Written informed consent was obtained from the patients for their study participation and for the publication of this report and any accompanying images. Patients with a history of instrumentation surgery, three-column osteotomy or Ponte osteotomy, follow-up for less than 24 months, and insufficient radiographic data were excluded. The following parameters were examined: number of vertebral bodies fixed, upper instrumented vertebra (UIV), type of CI, average number of segments subjected to LLIF, average volume of blood loss, operative time, various spinopelvic parameters, pre- and postoperative Oswestry Disability Index (ODI) and perioperative complications. The ODI was used to assess disability in all patients preoperatively and at 12 and 24 months postoperatively. CI was divided into two types based on the Obeid-coronal classification: convex CI that represents a CI in which the C7 plumb line-central sacrum vertical line (C7-CSVL) shifts to the convex side of scoliosis, and concave CI that refers to CI wherein the C7-CSVL shifts to the concave side of scoliosis [21]. We defined the distance between the spinous process of the UIV and the central sacrum vertical line (CSVL) as the UIV-CSVL (Figure 1). Patients were stratified based on UIV-CSVL after the first intraoperative rod application into groups P (UIV-CSVL > 10 mm, *n* = 50) and group G (UIV-CSVL < 10 mm, *n* = 65). The following measures were undertaken when the intraoperative UIV-CSVL was 10 mm or greater after first rod application: (1) rod rotation (RR), (2) S2 alar-iliac screw (SAI) distraction (SD), and (3) kickstand-rod technique (KR) [22] (Figure 2). RR is a correction method where the lumbar lordosis of the rods is converted into scoliosis by rotating the two main rods, maintaining the main curve, and thereby shifting the coronal balance (Figure 3). SD entails tilting the coronal balance toward the opposite side by lifting the main rod to the side where the coronal balance is tilted cranially, using the SAI screw head as a fulcrum (Figure 4). As reported previously, the KR correction method entails shifting the coronal balance to the opposite side by shifting the main rod to the cranial side using the additional rod connected to the additional iliac screw as a fulcrum [22]. We performed RR first, followed by SD or KR.

### 2.1. Radiological Evaluation

**Figure 5:**
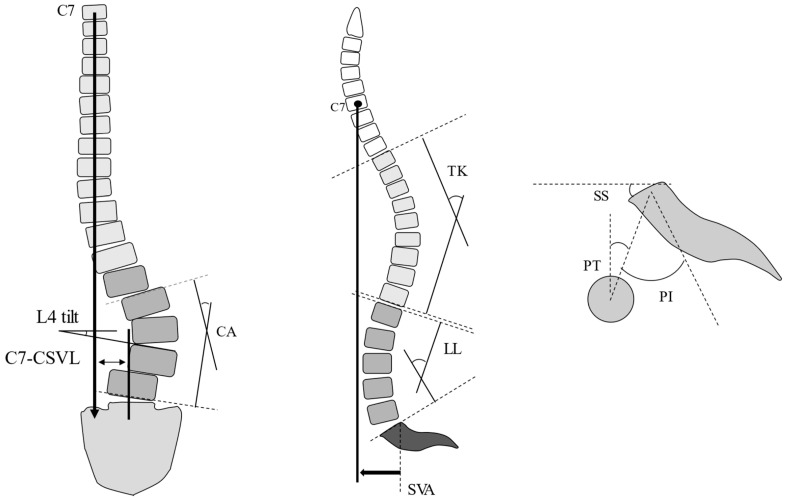
Spinopelvic parameters. L4 tilt: angle between the superior endplate and the horizontal. CA: Cobb angle; CSVL: central sacrum vertical line; LL: lumbar lordosis; PI: pelvic incidence; PT: pelvic tilt; SS: sacral slope; SVA: sagittal vertical axis; TK: thoracic kyphosis.

Standing posteroanterior and lateral whole-spine radiography was acquired at baseline (preoperatively) and final follow-up. The following spinopelvic parameters were investigated using the current standard methods: PI, LL, pelvic tilt (PT), thoracic kyphosis, sagittal vertical axis, CA, L4 tilt, and C7-CSVL (Figure 5). The L4 tilt angle was measured between the superior endplate and the horizontal. Proximal junctional kyphosis was defined as the postoperative proximal junctional angle between the caudal end plate of the UIV and the cephalad end plate of the UIV + 2 ≥ 10° and at least 10° greater than the preoperative measurements [23]. CI was defined as C7-CSVL ≥ 30 mm [9].

### 2.2. Statistical Analysis

Continuous variables were presented as the mean and standard deviation. The radiographic and clinical parameters were analyzed using the Wilcoxon signed-rank test for continuous variables, and the chi-squared test was used for categorical data. Statistical significance was set at *p* < 0.05. All analyses were performed using JMP pro 16 (SAS Institute Inc., Cary, NC, USA).

## 3. Results

Table 1 shows the participants’ demographic data. The patient background did not differ significantly between the two groups (Table 1). Preoperatively, convex CM was significantly more common in group P. The type of postoperative CI did not differ significantly between the two groups (Table 2). The preoperative and postoperative L4 tilt was significantly higher in group P than in group G. There was no significant difference in the other parameters (Table 3). Group P was further divided into three groups: group RR, wherein 38 patients underwent RR; group SD, wherein seven patients underwent RR and SD and group KR, wherein five patients underwent RR and KR. The ΔUIV-CSVLs were 13.9 mm, 20.1 mm, and 24.4 mm in these three groups, respectively. The greatest degree of CI correction was achieved with KR(RR and KR) and the least with RR. Postoperative C7-CSVL < 10 mm was achieved in all three correction groups (Table 4).

### 3.1. Case Study 1: RR

**Figure 6:**
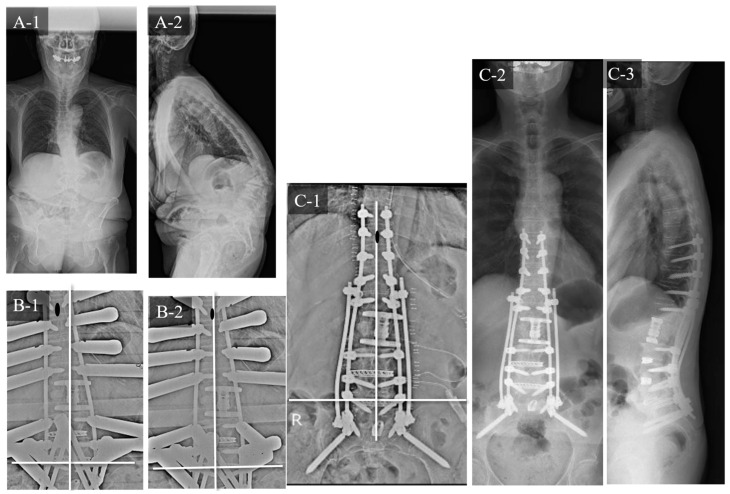
Case study 1 (RR). (**A-1**) Preoperative standing whole spine radiograph (AP). (**A-2**) Preoperative standing lateral whole spine radiograph. (**B-1**) Intraoperative radiograph before RR. (**B-2**) Intraoperative radiograph after RR. (**C-1**) Postoperative radiograph (AP). (**C-2**) Postoperative standing whole spine radiograph (AP). (**C-3**) Postoperative standing lateral whole spine radiograph. RR: rod rotation.

A 65-year-old woman (Figure 6) with ASD and an osteoporotic vertebral fracture at L2 exhibited the following preoperative spinopelvic parameters: PI, 47°; LL, −42°; PI-LL, 89°; PT; 48° and C7-CSVL, 5 mm. Initially, LLIF and lateral access corpectomy were performed, followed by PPS fixation from T9 to the pelvis and transforaminal lumbar interbody fusion (TLIF) at L5/S1 1 week later. Since the UIV-CSVL was shifted to the left side by 19 mm after the first application of the rod, RR was performed. Thereafter, the UIV-CSVL improved to 7 mm. Good coronal alignment was obtained after the procedure, and the postoperative C7-CSVL was less than 10 mm. Ideal alignment was maintained 24 months postoperatively.

### 3.2. Case Study 2: RR and SD

**Figure 7:**
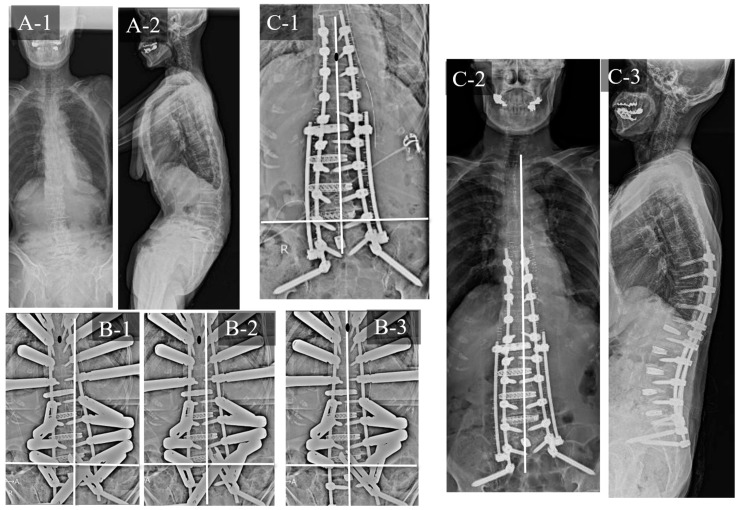
Case study 2 (RR and SD). (**A-1**) Preoperative standing whole spine radiograph (AP). (**A-2**) Preoperative standing lateral whole spine radiograph. (**B-1**) Intraoperative radiograph before RR. (**B-2**) Intraoperative radiograph after RR. (**B-3**) Intraoperative radiograph after SD. (**C-1**) Postoperative radiograph (AP). (**C-2**) Postoperative standing whole spine radiograph (AP). (**C-3**) Postoperative standing lateral whole spine radiograph. RR: rod rotation, SD: sacral alar-iliac screw distraction technique.

An 85-year-old woman with ASD (Figure 7) exhibited the following preoperative spinopelvic parameters: PI, 48°; LL, −21°; PI-LL, 69°; PT, 63° and C7-CSVL, 39 mm. Initially, LLIF was performed at L1/2 to L4/5, followed by PPS fixation from T9 to the pelvis and TLIF at L5/S1 1 week later. The UIV-CSVL was shifted to the right side by 41 mm after the first application of the rod. Therefore, RR was performed first, which improved the UIV-CSVL to 28 mm; however, the UIV-CSVL still exceeded 10 mm. Thereafter, SD was performed. The UIV-CSVL improved to 3 mm. Good coronal alignment was obtained after the procedure, and the postoperative C7-CSVL was 21 mm.

### 3.3. Case Study 3: RR and KR

**Figure 8:**
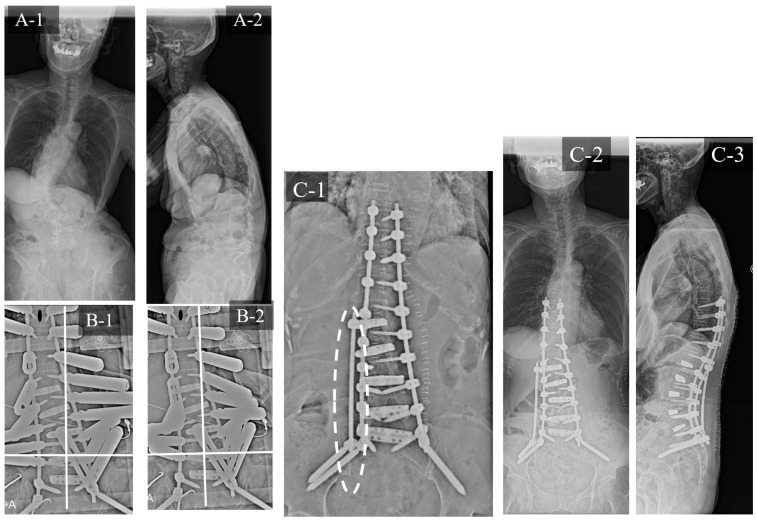
Case study 3 (RR and KR). (**A-1**) Preoperative standing whole spine radiograph (AP). (**A-2**) Preoperative standing lateral whole spine radiograph. (**B-1**) Intraoperative radiograph before RR. (**B-2**) Intraoperative radiograph after RR. (**C-1**) Intraoperative radiograph after KR. (**C-2**) Postoperative standing whole spine radiograph (AP). (**C-3**) Postoperative standing lateral whole spine radiograph. RR: rod rotation, KR: kickstand rod technique.

A 79-year-old woman with ASD (Figure 8) exhibited the following preoperative spinopelvic parameters: PI, 54°; LL, 0°; PI-LL, 54°; PT, 48° and C7-CSVL, 18 mm. Initially, LLIF was performed at L1/2 to L5/S1, followed by PPS fixation from T9 to the pelvis 1 week later. The UIV-CSVL was shifted to the right side by 34 mm after the first application of the rod. Therefore, RR was performed first, which improved the UIV-CSVL to 25 mm; however, the UIV-CSVL still exceeded 10 mm. Thereafter, KR was performed. The UIV-CSVL improved to 4 mm. Good coronal alignment was obtained after the procedure, and the postoperative C7-CSVL was 5 mm.

## 4. Discussion

Previous studies have designated CI as a form of coronal malalignment [21,24,25]. Numerous studies have reported the definition of CI after ASD surgery, such as postoperative C7-CSVL > 25 mm, 30 mm, or 40 mm [19,21,26,27,28]. The preoperative rate of CI in patients with ASD reportedly ranges from 19–35% [26,29,30], and the frequency of the latter has been reported to be 15–30% [20,26,29,30,31]. In this study, the preoperative rate of CI was 37%, while the postoperative incidence rate of CI was 4%, which was significantly lower than that reported by previous studies despite the use of CMIS, confirming the effectiveness of various measures against CI. The presence of preoperative CI exerts a tremendous influence on the incidence of postoperative CI [32]. Similar results were obtained in this study. That is, the preoperative CI rate was higher in Group P than in Group G. Surgical correction of CI can be difficult, and recent reports indicate that insufficient correction of CI is not negligible in ASD surgery [20,26,29,30]. CI has a lesser impact on pain and function than sagittal imbalance [24]; however, unlike sagittal imbalance, compensation by the spine and pelvis is difficult, necessitating precise intraoperative correction [21]. Flexion of the contralateral hip and knee can compensate for CI but is uncomfortable for the patient [21]. Tanaka et al. reported the relationship between postoperative CI and rod breakage, suggesting that the load on the rod due to CI affects rod breakage [14]. Therefore, we need to accurately evaluate and adjust coronal balance intraoperatively to prevent rod breakage after ASD surgery. Furthermore, Buell et al. reported that postoperative coronal imbalance had a negative impact on patient satisfaction [33]. Therefore, we should accurately correct not only sagittal alignment but also coronal alignment in ASD surgery.

### 4.1. Risk Factors for CI

Lewis et al. reported that postoperative L4 or L5 tilt is a risk factor for postoperative CI [20]. Zhang et al. and Tanaka et al. respectively reported that L4 tilt and L5 tilt are predictors of postoperative CI [14,34]. Matsumura et al. reported that inadequate correction of the lumbosacral fractional curve was a risk factor for postoperative CI [19]. The existence of L4 or L5 tilt can be considered to indicate the under-correction of the fractional curve, and the results of previous studies are generally similar. In this study, the preoperative and postoperative L4 tilt in group P was significantly larger than that in group G, which bore a close resemblance to the results of previous studies. Zhang J et al. and Zhang Z et al. also reported that convex CM is a risk factor for postoperative CI [34,35]. Moreover, in this study, the preoperative convex CM was significantly higher than the concave CM in group P. Hence, predicting the risk of CI occurrence and ascertaining whether the main curve or fractional curve requires correction before surgery are crucial.

### 4.2. Measures for CI

The importance of CI and sagittal imbalance has gradually received recognition, and various measures against CI have been reported. Obeid et al. devised a surgical algorithm for CI in spinal deformity, highlighting the importance of correction of the main curve in concave CM and correction of the lumbosacral fractional curve in convex CM [21]. Bao et al. reported that correcting the main curve and fractional curve in two stages reduced the incidence of postoperative CI to 4.7% [36]. The most important aspect of CI reduction in corrective surgery for ASD is the correction of the L4 and L5 tilt via sufficient intervertebral release at L4/L5/S1. Furthermore, accurate intraoperative C7-CSVL measurements and various countermeasures are important. However, obtaining extensive radiographs to accurately evaluate the C7-CSVL is difficult during surgery. Therefore, UIV-CSVL is used to evaluate coronal balance. The distance between C7 and S1 is less than twice the distance between T10 and S1. Therefore, it can be expected that C7CSVL can be adjusted to 20 mm or less by adjusting UIV-CSVL < 10 mm. Therefore, we used the UIV-CSVL as an intraoperative evaluation tool for coronal balance in ASD surgery. Kurra et al. reported that the use of intraoperative T-bars significantly reduced the postoperative CI [36]. The KR technique used in this study was first described in a case report by Makhni et al. in 2018 [22]. Other studies have also demonstrated its usefulness [25,37,38,39]. However, the measures used in this study, such as RR and SD, have not been described in previous studies. Redaelli et al. reported a “tie rod” procedure similar to the KR technique [25]. The KR technique entails shifting the coronal balance to the opposite side using distraction force generated via the additional rod connected to the additional iliac screw as the fulcrum; however, the principle of the “tie rod” method involves shifting the coronal balance to the side where the additional rod is installed with compression force between the additional rod and main rod. In this study, the correction force generated in the KR technique was stronger than that in the other two procedures, clarifying its utility (Table 3). However, as mentioned above, the disadvantage of the KR procedure is that it is more time-consuming than RR and SD because it requires additional instrumentation. We recommend performing the simplest procedure, i.e., RR first, followed by SD, and finally KR, as needed. The three methods introduced in this study function according to different correction principles. The RR correction method entails transforming the lumbar lordosis of the rods into scoliosis by rotating the two main rods; thus, the main curve remains, and the coronal balance is corrected. However, the SD method entails a correction of the fractional curve. In SD, it is possible to distract the intervertebral segments to facilitate correction over a wider range by increasing the number of parts where the set screw is loosened. The KR method functions by correcting both the global balance and fractional curve. The set screw of the segment that is intended to be opened is loosened and lifted to the opposite side using a kickstand. The decision to correct a wide range of instrumentation or only fractional curves can be changed depending on the case. RR is the simplest method, but there is a limit to its corrective force, while KR is time-consuming to install additional iliac screws and rods; however, KR generates a strong correction force. The SD procedure is simple and generates a strong correction force. Therefore, we generally perform RR first, followed by SD, and finally KR (Table 5).

The disadvantage of the RR procedure is that conversion of LL to scoliosis can reduce lordosis. RR was the most commonly used measure in this study; however, we could not confirm the case where LL was reduced using RR. This may be attributed to the fact that the RR angle provided by the rod holder used in CMIS is small, and the reduction in LL is minuscule.

The disadvantage of the SD procedure is that distraction of the intervertebral space results in the loss of contact between the cage inserted into the intervertebral space and the end plate, resulting in union failure.

The rationale for bony union is as follows. First, it may be achieved by the addition of distraction; the TLIF cage is used as a fulcrum, resulting in the narrowing of the opposite side, thereby stabilizing the interbody space. The second reason is that an additional rod was attached in all cases, which may have prevented rod fractures and resulted in the bony union. In any case, it would be possible to prevent poor contact between the cage and the end plates by subjecting several disc spaces to distraction, instead of just one disc space.

The disadvantage of the KR procedure is the reduction of LL by the addition of a distraction force [25]. Redaelli et al. reported that a “tie rod” is preferable for increasing LL, whereas the KR technique is suitable for the reduction of LL. This is an important consideration while performing corrective surgery for spinal deformity [25].

The correction of CI using the above-mentioned measures is considered difficult for interbody fusion at L4/5/S since fractional curve correction with SD and KR is difficult in such cases. However, cases in which both intervertebral segments are fused are extremely rare. If only one of the segments is fused, the effect of SD and KR can be expected in the non-fused segment. In addition, in the case of intervertebral fusion at L4/5, this measure is indicated because LLIF achieves intervertebral release in most cases. If interbody fusion in the lumbosacral spine contributes to sagittal misalignment, three-column osteotomy (3CO) is indicated and CMIS is contraindicated. Furthermore, these measures are implemented after the flexibility of the lumbosacral spine is restored by 3CO.

### 4.3. Limitations

There are some limitations to this study. The first arises from its retrospective design. Second, only the ODI was used to evaluate the health-related quality of life. Third, the sample size was relatively small, and the study was conducted at a single institution. The effectiveness of these measures should be further clarified by conducting a multicenter study with a larger sample size in the future. The fourth limitation is our evaluation method. Coronal balance was evaluated using the UIV-CSVL instead of the C7-CSVL. We should avoid using the UIV-CSVL in patients with scoliosis with vertebral body rotation or double-curve scoliosis in the thoracic spine when assessing intraoperative coronal balance. However, since most ASD patients mainly have lumbosacral deformity, we can use UIV-CSVL as an intraoperative evaluation tool for coronal balance. Another methodological limitation is that the CSVL is measured as a perpendicular line connecting the iliac crests on both sides. Accurate evaluation of global coronal balance is difficult when the iliac crest is used as a reference in patients with osteoarthritis of the knee and hip joints and differences in leg length. In these patients, it is necessary to evaluate the coronal balance from the head to the toes; however, in most cases, an accurate evaluation of the coronal balance is possible with our evaluation method. The sixth limitation is that the flexibility of the coronal plane was not evaluated using side bending before surgery. Since the flexibility of the coronal and sagittal planes changes significantly with LLIF, we do not place importance on the preoperative evaluation of flexibility. In the majority of patients with intervertebral fusion, flexibility changes significantly after surgery, since intervertebral release is achieved with LLIF [15]. However, the possibility of a correlation between preoperative and post-LLIF flexibility of the coronal plane and the effect of this measure cannot be completely dismissed, and detailed verification is required in the future.

## 5. Conclusions

We introduced measures and described their effects on CI after CMIS for ASD. The preoperative L4 tilt and the preoperative convex CI rate were significantly higher in group P than in group G. The measures reported in this study are useful for the prevention of postoperative CI. Implementing RR as the first measure, followed by SD, and finally KR, facilitates easy and efficient correction of coronal balance after CMIS for ASD.

## Figures and Tables

**Table 1:** Demographic data.

Parameter		Group P (*n* = 50)	Group G (*n* = 65)	*p* Value (Group P vs. Group G)
Age (years)		73.3 ± 6.9	73.0 ± 7.7	0.762
Rate of women (%)		80.8	78.1	0.859
Period of follow-up (months)		40.7 ± 6.3	40.6 ± 6.2	0.792
Rod diameter/number in construct	5.5 mm/2 rods	20	25	0.509
	6 mm/2 rods	10	13	
	5.5 mm/3 rods	20	27	
Number of levels fused		10.3 ± 0.5	10.4 ± 0.5	0.721
Number of LLIF		4.0 ± 0.3	4.1 ± 0.5	0.692
UIV (case)	T8	2	0	0.185
	T9	15	16	
	T10	33	49	
Operative time (min)	Anterior (first surgery)	112.4 ± 40.2	100.8 ± 26.5	0.613
	Posterior (second surgery)	233.2 ± 52.1 **	233.0 ± 47.7 **	0.752
Blood loss (mL)	Anterior (first surgery)	117.7 ± 152.4	63.0 ± 75.1	0.316
	Posterior (second surgery)	488.6 ± 290.9 **	528.1 ± 350.9 **	0.263
ODI	before surgery	41.3 ± 6.2	40.1 ± 5.1	0.425
	Final	24.8 ± 5.8 *	25.9 ± 2.6 *	0.501
Complications	PJK	12%	15%	0.679
	RF	9%	13%	0.498

Values are presented as mean ± standard deviation. * Statistically significant, Wilcoxon signed-rank test or chi-squared test. UIV: upper instrumented vertebra, LLIF: lateral lumbar interbody fusion, VAS: visual analog scale, ODI: Oswestry Disability Index, PJK: proximal junctional kyphosis, RF: rod fractures. * *p* < 0.001 compared with before surgery, ** *p* < 0.001 compared with the first surgery.

**Table 2:** Type of CI.

Type of CI			Total (*n* = 115)	Group P (*n* = 50)	Group G (*n* = 65)	*p* Value (Concave CI vs. Convex CI)
Preop	Non-CI (case)		72 (63%)	26	46	0.031 *
	CI (case)		43 (37%)	24	19	
	CI	Concave CI (case)	23 (20%)	10	14	0.036 *
		Convex CI (case)	20 (17%)	14	5	
Postop	Non-CI (case)		110 (96%)	47	63	0.377
	CI (case)		5 (4%)	3	2	
	CI	Concave CI (case)	2 (1%)	0	2	0.100
		Convex CI (case)	3 (3%)	3	0	

* Statistically significant, chi-squared test. CI: coronal imbalance.

**Table 3:** Various parameters (group P vs. group G).

	Group P (*n* = 50)	Group G (*n* = 65)	*p* Value
PI (°)	43.1 ± 10.5	46.5 ± 10.8	0.071
Preop LL (°)	9.2 ± 16.1	13.0 ± 15.8	0.258
Postop LL (°)	46.2 ± 10.2	48.8 ± 10.6	0.253
Preop PI-LL (°)	33.9 ± 17.9	38.5 ± 18.1	0.186
Postop PI-LL (°)	1.2 ± 11.9	2.6 ± 12.1	0.142
Preop PT (°)	29.5 ± 11.1	30.7 ± 11.3	0.592
Postop PT (°)	16.9 ± 9.8	18.9 ± 9.9	0.089
Preop TK (°)	21.3 ± 16.2	16.2 ± 16.3	0.123
Postop TK (°)	38.8 ± 10.9	36.7 ± 11.1	0.355
Preop C7CSVL (mm)	45.4 ± 36.9	40.1 ± 34.1	0.166
Postop C7CSVL (mm)	8.0 ± 8.1	6.2 ± 7.2	0.142
Preop CA (°)	42.3 ± 32.9	39.1 ± 31.0	0.326
Postop CA (°)	16.2 ± 9.0	12.2 ± 8.1	0.162
Preop L4 tilt (°)	13.2 ± 8.8	7.2 ± 6.9	0.026 *
Postop L4 tilt (°)	5.3 ± 4.1	2.1 ± 2.3	0.042 *

Values are presented as mean ± standard deviations. * Statistically significant, Mann–Whitney U test. L4 tilt: angle between the superior endplate and the horizontal. PI: pelvic incidence; LL: lumbar lordosis; PT: pelvic tilt; TK: thoracic kyphosis; SVA: sagittal vertical axis; CA: Cobb angle; C7PL-CSVL: C7 plumb line-central sacrum vertical line; UIV: upper instrumented vertebra.

**Table 4:** Parameters for each measure.

		Group RR (*n* = 38)	Group SD (RR and SD) (*n* = 7)	Group KR (RR and KR) (*n* = 5)	Total
UIV-CSVL	Pre-measure (mm)	19.9 ± 17.5	25.3 ± 3.9	32.1 ± 1.4	24.8 ± 5.9
	Post-measure (mm)	6.2 ± 1.7	7.2 ± 1.8	7.7 ± 0.8	10.1 ± 2.2
	ΔUIV-CSVL (mm)	13.9 ± 2.1	20.1 ± 1.9	24.4 ± 1.1	14.7 ± 4.2
C7-CSVL	Preop (mm)	39.2 ± 9.9	34.1 ± 9.1	35.5 ± 5.1	38.1 ± 9.8
	Postop (mm)	7.2 ± 1.8	9.2 ± 1.9	8.5 ± 1.2	8.0 ± 1.8

Values are presented as mean ± standard deviations. UIV-CSVL: upper instrumented vertebra-central sacrum vertical line, C7-CSVL: C7 plumb line-center sacrum vertical line, RR: rod rotation technique, SD: S2 alar iliac screw distraction technique, KR: kickstand rod technique.

**Table 5:** Measures for CI in CMIS.

	Principle of Correction	Correction Force	Limitation of Correction	Required Time
RR	Global imbalance	weak	limited	2 or 3 min
SD	Correction of lumbosacral fractional curve	moderate	limited	About 5 min
KR	Correction of lumbosacral and global imbalance	strong	limited	More than 10 min

CI: coronal imbalance; CMIS: circumferential minimally invasive correction surgery; RR: rod rotation technique; SD: S2 alar iliac screw distraction technique; KR: kickstand rod technique.

## Data Availability

The datasets used and/or analyzed during the current study are available from the corresponding author upon reasonable request.
